# Histone Deacetylases (HDACs) and Atherosclerosis: A Mechanistic and Pharmacological Review

**DOI:** 10.3389/fcell.2020.581015

**Published:** 2020-11-12

**Authors:** Xiaona Chen, Yanhong He, Wenjun Fu, Amirhossein Sahebkar, Yuhui Tan, Suowen Xu, Hong Li

**Affiliations:** ^1^Department of Medical Biotechnology, School of Basic Medical Sciences, Guangzhou University of Chinese Medicine, Guangzhou, China; ^2^The Research Center of Basic Integrative Medicine, Guangzhou University of Chinese Medicine, Guangzhou, China; ^3^Biotechnology Research Center, Pharmaceutical Technology Institute, Mashhad University of Medical Sciences, Mashhad, Iran; ^4^Neurogenic Inflammation Research Center, Mashhad University of Medical Sciences, Mashhad, Iran; ^5^Polish Mother’s Memorial Hospital Research Institute, Łódź, Poland; ^6^Department of Endocrinology, First Affiliated Hospital, Division of Life Sciences and Medicine, University of Science and Technology of China, Hefei, China

**Keywords:** atherosclerosis, endothelial dysfunction, smooth muscle cells, macrophage, epigenetic, histone deacetylation, HDAC inhibitors

## Abstract

Atherosclerosis (AS), the most common underlying pathology for coronary artery disease, is a chronic inflammatory, proliferative disease in large- and medium-sized arteries. The vascular endothelium is important for maintaining vascular health. Endothelial dysfunction is a critical early event leading to AS, which is a major risk factor for stroke and myocardial infarction. Accumulating evidence has suggested the critical roles of histone deacetylases (HDACs) in regulating vascular cell homeostasis and AS. The purpose of this review is to present an updated view on the roles of HDACs (Class I, Class II, Class IV) and HDAC inhibitors in vascular dysfunction and AS. We also elaborate on the novel therapeutic targets and agents in atherosclerotic cardiovascular diseases.

## Introduction

Atherosclerosis (AS) is the critical underlying pathology of CVD, which ranks the first on the morbidity and mortality of diseases (Fanelli et al., 2017; Libby et al., 2019; Niu et al., 2019; Wang et al., 2019). It lessens the elasticity of the arteries and may lead to myocardial infraction, ischemic stroke, cerebrovascular incidents, and peripheral vascular disease (Ziegler et al., 2019). The most prominent characteristic of AS is plaque formation in the arteries. Although the cause of the spontaneous AS and its initially characteristic focal plaque morphology has not been well understood, the histology and the progression of the advanced plaque have been identified (Ross, 1993; Poston, 2019). The progression of AS includes low-density lipoprotein (LDL) oxidation, endothelial activation, monocytes recruitment, macrophage-derived foam cell formation, VSMC proliferation, and thrombus formation (Poston, 2019).

Among the multiple mechanisms that exist in the development of AS, endothelial dysfunction has been recognized as one of the major cardiovascular risk factors (Arcaro et al., 1995). The healthy endothelium is important to maintain vascular homeostasis. It possesses the function of generating bioactive NO, regulating vascular tone, protecting the endothelial cell (EC) integrity, repairing the injury and inducing angiogenesis (Huynh and Heo, 2019). However, impaired NO bioavailability, oxidative stress, inflammation cytokines, and vascular tone potentially disrupt the endothelium homeostasis with consequence of endothelial dysfunction. Once the endothelium function is altered, followed by increased permeability to lipoprotein that attracts more leukocytes, induced secretion of inflammation cytokines and ROS, but less NO production (Poston, 2019); it exacerbates the pathology lesion of the arteries. Ensuing events of endothelial dysfunction include the proliferation and migration of VSMCs and formation of foam cell (Tian K. et al., 2019).

The epigenetic modification on genes is a crucial mechanism for many diseases, including cancer and CVDs (Xu et al., 2018, 2019; Khan et al., 2020). As one of histone modifications, histone acetylation plays an important role in altering the condensation of chromatin (which is mainly composed of DNA and histones in the nucleus of cells) without changing DNA sequences and has been regarded as the potential therapeutic targets. Acetylation of histones and nonhistone proteins is achieved by histone acetylases but removed by HDACs, which can regulate the transcriptional activities of the specific genes via interaction with the histones and transcription factors (Shirodkar and Marsden, 2011). In fact, the role of HDACs in cancer has been extensively studied *in vivo* and *in vitro*. Particularly, there are several HDAC inhibitors (HDACi) that have been approved by the Food and Drug Administration (FDA) for clinical application in cancer. During last two decades, much attention has been focused on the critical involvement of HDACs in CVD. The purpose of this article is to provide a systematic review on the role of HDACs and their inhibitors in vascular function and the progression of AS and highlight the potential application of HDACi in treating AS.

## Histone Deacetylases

**FIGURE 1:**
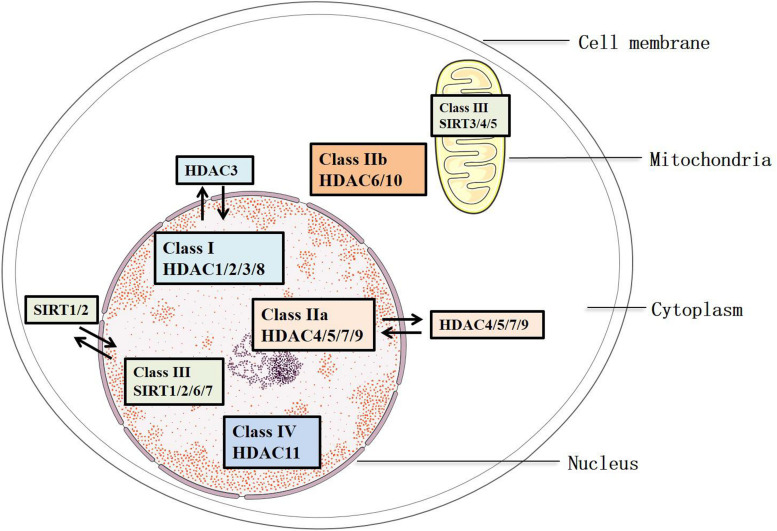
Classification and sublocation of HDACs.

Histone deacetylases remove the acetylated residues at lysine, redense the chromatin structures, and inhibit the transcription of target genes (Bae et al., 2009). They are divided into two families, HDAC family and sirtuin family, including 18 members. These members are characterized into four groups: Classes I, II, III, and IV (Parra and Verdin, 2010) (Figure 1). Class III consists of the sirtuin family (SIRT1-7), which has been identified since 21st century. They differ from other groups because of their specific conserved catalytic core domain that requires the binding of NAD^+^/NADH, whereas the others require zinc molecule as an activator (Mihaylova and Shaw, 2013). Correspondingly, HDACs in Classes I, II, and IV, other than Class III, are referred to as the classical HDACs. Class I (HDAC1/2/3/8) HDACs are similar to the yeast Rpd3 and mostly locate in the nucleus, except that HDAC3 can export to the cytoplasm. In addition, Class II HDACs were subclassified into Classes IIa and IIb based on their primary structures. Class IIa (HDAC4/5/7/9) subfamily contains only an N-terminal regulatory domain, whereas Class IIb (HDAC6/10) subfamily has two catalytic domains (Gray and Ekström, 2001). All Class IIa members shuttle between the nucleus and the cytoplasm, which interact with the kinase families such as the calcium-independent protein kinase and the MAPK, acting as a signal transducer, but Class IIb (HDAC6/10) is mainly located in the cytoplasm (Fischle et al., 2002). As for Class III, SIRT1/2 are located in both the nucleus and cytoplasm, SIRT6/7 are in the nucleus, and SIRT 3/4/5 are in the mitochondria (Luo et al., 2014) (Figure 1). Of note, the location of HDACs might vary from different types of cells. HDAC11 is the only member of Class IV, and it is a negative regulator of interleukin (IL) 10 and the activity of T cells, indicating the potential role of HDAC11 in treating AS, which occurs as an inflammatory process (Yanginlar and Logie, 2018).

Although HDACs were primarily identified as enzymes that deacetylate histones, further studies have identified many other nonhistone protein substrates, especially transcription factors such as the p65 subunit of NF-κB, E2F1, SP1, KLF 2/4, and signal transducers and activators of transcription 1 (STAT1) (Lyu et al., 2019). Because of multifunctional properties of HDACs, they have been involved in many cellular activities, tissue development, and various diseases, including embryonic development, tissue function, viral infections, CVD, cancer, kidney diseases, and autoimmune diseases (Gatla et al., 2019). Of note, the sirtuin family is well known for their effects in regulating vascular health, which have been deeply analyzed in recent reviews (Kane and Sinclair, 2018; Zhang et al., 2020). Hence, in this review, we mainly focus the role of classical HDACs in vascular function and AS, as well as the pharmacological effects of HDACi on AS.

## HDAC Inhibitors

Because of the fact that HDACs are implicated in triggering the development of some diseases, especially cancer, HDACi have been designed or investigated. These inhibitors mainly bind with the catalytic sites of HDACs, resulting in genes re-expression (Zhang et al., 2018). Although HDACi are specific for HDACs, most of them are not specific to the HADC subclass. Based on their chemical structures, HDACi can be classified into four groups: short-chain fatty acids, benzamides, hydroxamic acids, and cyclic peptides (Marks, 2010). To date, five HDACi (SAHA, LBH589, PXD101, VPA, romidepsin) have been approved by the FDA for clinical treatment of cancer (Gatla et al., 2019). In addition, some HDACi are also in clinical trials for other diseases such as human immunodeficiency virus infection (Elliott et al., 2014), sickle cell disease (Okam et al., 2015), Duchenne muscular dystrophy (Bettica et al., 2016), polycythemia vera (Finazzi et al., 2013), and myeloproliferative diseases (Rambaldi et al., 2010). Although currently there is still no clinical usage of HDACi in CVD, plenty of evidence has shown the great potential of some HDACi in inhibiting endothelial dysfunction and AS (Zheng et al., 2015; Xu et al., 2017; Lee and Chiu, 2019). Notably, as some HDACs protect vascular cells against injury triggered by proatherogenic stimuli, it is necessary to clarify the specific role of each HDAC in different cellular context and activities in the vasculature, so that HDACi could be better repurposed for cardiovascular therapeutics.

## Role of HDACs in Atherosclerosis

### HDACs Are Involved in Endothelial Function and Dysfunction

#### HDACs and NO Production

Nitric oxide is a catalytic product of endothelial NO synthase (eNOS) in the endothelium, with the L-arginine as the substrate and BH_4_ as the cofactor (Förstermann et al., 2017). NO production can be inhibited by arginase 2 or AngII from VSMCs (Krause et al., 2016; Ryu et al., 2019). Once NO diffuses across the EC membrane, it can activate the sGC rapidly in VSMCs. The activated sGC catalyzes GTP to generate cGMP, a second messenger mediating the PKC signaling pathway, which results in a decline of the intracellular concentration of calcium and triggers the vasodilation of the VSMCs (Feil et al., 2003). The endogenous NO not only decreases the vascular tone but also inhibits the progression of the inflammation and the angiogenesis. Interestingly, HDACs and their inhibitors have been found to modulate the production of NO.

On the one hand, HDACs regulate the expression of eNOS. For example, HDAC1 was recruited at eNOS promoters and impaired eNOS expression in ECs subject to ischemic/reperfusion insult, leading to decrease in NO formation, which was ameliorated by the HDACi TSA treatment (Yang et al., 2012). In ECs from DJ-1/park7^–/–^ mice, HDAC1 also inhibited eNOS transcription by inhibiting histone acetylation at the eNOS promoter (Won et al., 2014). HDAC3 (Zhang et al., 2008) and HDAC6 (Chen et al., 2019) had similar effects in inhibiting eNOS expression, and the effects could be reversed by tubacin, a selective inhibitor of HDAC6. By contrast, fluid shear stress induced phosphorylation of HDAC5, which was subsequently exported from nucleus and was involved in increased eNOS expression (Wang W. et al., 2010; Kwon et al., 2014). However, both of the HDACi-BuA and entinostat (MS-275) caused a decrease in eNOS protein (Rossig et al., 2002). In addition, the authors found that although TSA increased the eNOS promoter transcriptional activity, it reduced the production of NO through posttranscriptional suppression of eNOS protein levels. On the other hand, eNOS activity was regulated by HDACs via posttranslational modifications. HDAC1 (Hyndman et al., 2014) and HDAC3 (Jung et al., 2010) could reduce the lysine acetylation of eNOS and blocked NO expression. On the contrary, VPA could improve NO production by influencing eNOS phosphorylation (Cho et al., 2014). Of note, overexpression of HDAC2 could suppress the expression of arginase 2, a protein that counteracts eNOS activity, and the effect was reversed by TSA through increasing levels of H3K9 and H4K12 acetylation at arginase 2 proximal and core promoter (Pandey et al., 2014; Krause et al., 2016), indicating a protective role of HDAC2 in endothelial dysfunction and AS.

#### HDACs and Endothelial Oxidative Stress

BH_4_, the coactivator of the eNOS, can be easily oxidized to dihydrobiopterin, resulting in uncoupling of eNOS, leading to superoxide rather than NO production (Stocker and Keaney, 2004). Superoxide interacts with NO to produce ONOO^–^, which reduces the bioavailability of NO. In general, superoxide is mainly derived from the process when the membrane oxidase Nox transfers the electrons from NADPH to oxygen (Craige et al., 2015). Among the members of Nox family, Nox1/2/4/5 are expressed in the cardiovascular system with abundant expression of Nox4.

It was reported that the pan-HDACi varinostat (SAHA) reduced expression of Nox1/2/4 in the aorta of *ApoE*^–/–^ mice, which contributed to its anti-AS effect (Manea et al., 2020). Recruitment of HDAC abolishes the interaction of RNA polymerase II and p300 to the promoter sites of Nox2/4/5, respectively, which inhibited the activation at those promoter regions and resulted in a decline of ROS (Chen et al., 2016; Hakami et al., 2016). In addition, HDAC3 could ameliorate the oxidative stress induced by AngII or disturbed flow (Martin et al., 2014). In another aspect, HDAC1 and HDACi could regulate the antioxidant enzymes including SOD and CAT, which scavenge the superoxide *in vivo* or *in vitro*. HDAC1 dissociation from SOD3 promoter was critically involved in SOD3 expression elicited by caffeic acid phenethyl ester (Ohashi et al., 2017). It is reported that TSA could induce the expression of SOD3 and reduce NOX expression robustly in human pulmonary artery ECs exposed to scriptaid (Zelko and Folz, 2015). However, MS-275 failed to influence the expression of Nox1, Nox2, and p47phox (Ryu et al., 2019).

Reactive oxygen species are the stimuli that reduce the bioavailability of NO and induce inflammation of ECs. It was found that IGF-1 enhanced the phosphorylation of HDAC5 that is associated with AS and led to nuclear export of HDAC5, which was mediated by Nox4-dependent ROS production, as well as the phosphatidylinositol 3-kinase (PI3K)/AKT pathways (Pietruczuk et al., 2019). HDAC6 expression and activity were upregulated in ox-LDL-treated ECs, which led to decreased expression of cystathionine γ-lyase and contributed to endothelial dysfunction (Leucker et al., 2017), and knockdown of HDAC6 or pharmacological inhibition with the dietary HDACi sodium butyrate (Hou et al., 2018; Wu et al., 2018), the inhibitor of Class I HDAC, could inhibit endothelial dysfunction. Moreover, loss of HDAC2 or specific inhibition of HDAC6 was found to activate HO-1/SIRT1 pathway and inhibit oxidative stress induced by high glucose (Gao et al., 2018; Abouhish et al., 2020).

#### HDACs and Endothelial Inflammation

Oxidative stress, eNOS uncoupling, and inflammation are potential contributors to endothelial dysfunction. Inflammation in vasculature causes alteration of vascular wall, which can trigger the CVD especially AS (Ali et al., 2018). In the development of AS, ECs and macrophages produce the proinflammatory cytokines, such as TNF-α, IL-β, and IL-6 (Barbour and Turner, 2014). These proinflammatory molecules cause the secretion of adherent molecules, including ICAM-1, VCAM-1, and E-selectin, to recruit the leukocytes and monocytes to endothelium (Xu et al., 2017). TNF-α mediated an important signaling pathway in increasing the expression of inflammatory cytokines and superoxide, thereby attracting more monocytes into the subendothelium space (Kleinbongard et al., 2010). Accumulating studies have demonstrated that NF-κB plays a crucial role in promoting the inflammation cytokines release. Activation of NF-κB leads to the upregulation of TNF-α, IL-β, IL-6, and adherent molecules in ECs (Stein et al., 2010). In human ECs treated with ox-LDL, HDAC1 and HDAC2 were downregulated, and the effect was reversed by simvastatin, which also inhibited the NF-κB pathway (Dje N’Guessan et al., 2009). HDAC2 is also a mediator of neutrophil migration and regulated multiple MMPs and CD16 gene expression in acute ischemic stroke patients (Li et al., 2020). Similarly, phosphorylation of HDAC5 led to HDAC5 nuclear export and upregulation of KLF2 and mediated the anti-inflammatory effects of metformin in ECs (Tian R. et al., 2019). On the contrary, several HDACs were also implicated in the proinflammatory response in ECs. Bedenbender et al. (2019) reported that in TNF-α-treated human umbilical vein ECs (HUVECs), HDAC2 was recruited to the RNase I promoter and reduced the histone acetylation, leading to downregulation of RNase I, a protective molecule in vascular homeostasis. HDAC3 mediated the inflammatory response in ECs by regulating galectin-9 expression (Alam et al., 2011). In human pulmonary ECs subjected to *Staphylococcus aureus* infection, HDAC6 was upregulated followed by elevated ROS, and knockout or pharmacological inhibition of HDAC6 in mice could inhibit vascular inflammation and protect the EC integrity (Karki et al., 2019). It was suggested that the detrimental effect of HDAC6 might be mediated by microtube destabilization. Moreover, HDAC7 could induce leucocyte adhesion to ECs (Ismail et al., 2012), and HDAC8 was involved in increased secretion of ICAM-1 and VCAM-1 in the aortas of mice infused with AngII (Kee et al., 2019).

Because of the proinflammatory effects of most classical HDACs, several HDACi have been employed to determine their anti-inflammatory effects. In human lung ECs, LPS upregulated IKBα mRNA, which might be mediated by deacetylation of H3K9 and could be blocked by TSA (Thangjam et al., 2014). By contrast, TSA could suppress COX-2 expression induced by LPS through inhibiting the phosphorylation of JNK and p38 MAPK (Hsu et al., 2011). Moreover, TSA suppressed VCAM-1 (but not ICAM-1) expression in TNF-α-induced HUVECs and sickle transgenic mice, whereas MS-275 inhibited VCAM-1 and MCP-1 expression in AngII-induced hypertensive mice (Inoue et al., 2006; Hebbel et al., 2010; Ryu et al., 2019). He et al. (2011) showed that TSA inhibited the expression of TLR4 and HDAC2 induced by LPS in cultured EA.hy926 cells, which protected the EC from injury. Li et al. (2018a) found that TSA inhibited IL-8 production, and VCAM-1 expression induced by TNF-α in HUVECs, and the adhesion of peripheral blood mononuclear cell to HUVECs was also blocked. Besides, butyrate (Ogawa et al., 2003), other short-chain fatty acids (Miller et al., 2005; Vinolo et al., 2011), and HDAC8 selective inhibitor PCI34051 (Kee et al., 2019) also showed anti-inflammatory effect in ECs. It is notable that although HDACi exhibited an anti-inflammatory effect in various ECs or animal models, the inflammatory markers detected in each study varied, and the regulatory mechanism was also not well illustrated. Further study is needed to demonstrate the specific HDAC isoforms involved in and to evaluate whether the anti-inflammatory effect of HDACi is dependent on increased acetylation of histones or specific transcription/repressive factors.

#### HDACs Regulate EC Proliferation

Endothelium works as a selective barrier between the blood and tissue, controlling exchange of the ions and cytokines. Under healthy conditions, ECs tend to be tight and are not proliferating, but they start to migrate and proliferate when subjected to hypoxia, injury, or stress (Zeng et al., 2009). Although EC proliferation is required to response to the hypoxia, excessive EC proliferation contributes to EC turnover, which is critically associated with the endothelium permeability (Caplan and Schwartz, 1973).

β-Catenin is a signal transducer that accelerates cell proliferation and growth via activating the Id2, T cell factor/lymphoid enhancer factor, and follistatin transcription factors. Margariti et al. (2010) found that HDAC7 was important in modulating the expression of genes related to EC proliferation via regulating the transcription activity of β-catenin. Overexpression of HDAC7 suppressed HUVEC proliferation through inhibition of nuclear translocation of β-catenin and downregulation of Id2 and cyclin D1 expression, causing G1 phase elongation. The effect of HDAC7 overexpression could be abolished by the VEGF, which degraded HDAC7 via PLC-PI3K signal pathway and disrupted the complex of HDAC7 and β-catenin, leading to β-catenin released into the nucleus. It was shown that VEGF induced phosphorylation and cytoplasm translocation of HDAC7, resulting in activation of VEGF-responsive genes, and EC proliferation was enhanced (Wang et al., 2008). Similarly, HDAC4 and HDAC5 were also critical in mediating EC proliferation during cardiovascular development (Kang et al., 2013). However, in most cases, the classical HDACs were deemed to promote abnormal EC proliferation. Lee et al. (2012; Chiu and Chien, 2011) found that HDAC1/2/3-specific siRNAs reversed the increased level of cyclin A and reduction of p21 induced by OSS, which is considered to be a contributor to endothelial dysfunction of arterial branches and curvatures. Meanwhile, the HDACi VPA could suppress OSS-induced EC proliferation in BrdU-infused rats (Lee et al., 2012). HDAC4 and HDAC5 in nuclear could inhibit myocyte-enhancer factor 2 (MEF2) and KLF2/4 activity (Yang et al., 2019), whereas HDAC5 in cytoplasm mediated KLF2 expression (Wang W. et al., 2010; Kwon et al., 2014). Normal shear stress induced HDAC6 activity, which reduced tubulin acetylation and promoted ECs migration (Wang Y.H. et al., 2010). The pro-proliferation effect of VEGF could be inhibited by expressing a signal-resistant HDAC7 mutant protein in ECs (Wang et al., 2008). In parallel, the HDACi such as SAHA (Cheng and Hung, 2013), tubacin (Li et al., 2016), TSA, and apicidin (Yang et al., 2015) inhibited EC proliferation in different conditions.

#### HDACs and Endothelium Integrity

The endothelial barrier possesses tight junction, endothelial glycocalyx, and efflux transporters that are all essential for protecting the endothelial integrity and cell permeability and control the entry of the chemicals exchange between the blood and tissues. The endothelial glycocalyx is on the endothelial surface (Weinbaum et al., 2007). The MMPs are produced by ECs, which could degrade the ECM and connective tissue proteins, including the ones that form the glycocalyx (Haas, 2005). HDACs play a crucial role in protecting the endothelial barrier function by regulating cell permeability, chemical transporter, tight junction protein, and MMPs (Gao et al., 2008; Joshi et al., 2015; Shi et al., 2016; Castro et al., 2018). Among the classical HDACs, it seems that only HDAC7 and HDAC3 exhibit a protective effect in endothelial integrity. It was demonstrated that HDAC7 could interact with MEF2, inhibiting its transcription activity, and thus suppressed the expression of target genes such as MMP-10 (Chang et al., 2006; Gao et al., 2008; Su et al., 2013). By contrast, RGFP-966, an HDAC3-selective inhibitor, was found to significantly attenuate the oxygen–glucose deprivation/reperfusion-induced transendothelial cell permeability and downregulate the tight junction protein claudin-5 via activating PPARγ signaling pathway (Zhao et al., 2019). HDAC4 and HDAC5 regulated expression of connexin 37 and 40 in PAECs (Kim et al., 2015), and increased HDAC5 activity was essential for MMP induction, glycocalyx remodeling, and reduced expression of TIMP (Ali et al., 2019). As for HDAC6, it was a mediator leading to *S. aureus*-induced (Karki et al., 2019) or TNF-α-induced (Yu et al., 2016) endothelial permeability and could attenuate tubulin acetylation, leading to reduced cell stability (Fernandes et al., 2015). Besides, inhibition of HDAC6 could attenuate CSE-induced EC permeability and acute lung injury (Borgas et al., 2016). As the only member of class IV HDACs, HDAC11 is least studied in endothelial function. It was found that HDAC11 was upregulated in PAR2 agonist-treated ECs and mediated the impaired barrier function via modulating VE-cadherin expression (Zhang and Ge, 2017). Moreover, inhibition of HDACs via TSA or SAHA increased the protein levels of TIMP-1 and TIMP-3 (Ali et al., 2019).

The multidrug resistance protein 1 (also known as MDR1) is a crucial efflux transporter located on the top surface of capillary ECs and prevents xenobiotics accumulating in the brain (Serlin et al., 2015). You et al. (2019) showed that VPA, apicidin, and SAHA could increase MDR1 expression in human brain ECs. Glucose transporters are responsible for the energetic supply at the blood–brain barrier (Castro et al., 2016). β-Hydroxybutyrate, an HDACi, could upregulate the glucose transporter gene Slc2a1 expression in brain microvascular EC s and NB2a neuronal cells by increasing acetylation of H3K9 at the promoter site of the Slc2a1 gene (Rafehi et al., 2017). Besides, TSA, VPA, short-chain fatty acid, and the specific Class IIa HDACs inhibitor TMP269 could reverse endothelial barrier dysfunction induced by hemorrhage, lethal scald injury, and acute lung injury, respectively (Miyoshi et al., 2008; Bruhn et al., 2018; Tang et al., 2018; Kovacs-Kasa et al., 2020).

#### HDACs and Endothelial Apoptosis

Endothelial cell apoptosis is not active under healthy conditions, but it is activated in the development of AS and contributes to the hyperpermeability of endothelium, as well as thrombus formation on eroded plaques (Tedgui and Mallat, 2003). Li et al. (2018c) found that HDAC1 was reduced in AS lesions and ox-LDL-treated human aortic ECs (HAECs), which was regulated by miR-34a and might mediate EC apoptosis. In the process of senescence induced by radiation, HDAC1 was also reduced (Okamoto et al., 2006). Similarly, HDAC3 is critical for endothelial survival, and knockdown of HDAC3 led to apoptosis in ECs (Zampetaki et al., 2010). However, HDAC6 was implicated in apoptotic response of lung ECs induced by TNF-α (Yu et al., 2016). VPA induced the Bcl-2 phosphorylation and release of the cytochrome c via activating ERK1/2 and consequently inhibited the serum starvation-induced HUVECs apoptosis (Michaelis et al., 2006). Comparatively, VPA was found to induce apoptosis of tumor cells (Yamanegi et al., 2015).

#### HDACs and Angiogenesis

Angiogenesis mainly refers to the formation of functional capillaries especially in the progression of tumor, and it is a complex biological process including alterations of gene expression. Numerous studies were focused on the role of HDAC/HDACi and angiogenesis in cancer, which have been reviewed elsewhere (Mastoraki et al., 2020). Actually, the angiogenic ability of EC is also very important for maintaining the cell or tissue function during development, ischemia, hypoxia, or injury. VEGF is considered as a predominant growth factor during the formation of capillaries, which has been a therapeutic target for tumor angiogenesis (Pepper et al., 1996). Notably, VEGF could be induced by HIF-1α activation in response to hypoxia (Ikeda, 2005). Inhibition of HDAC1/4/6/7 abolished the expression of VEGF via impairing the induction or activity of HIF-1α in hypoxia-exposed ECV304 cells, HaCaT cells, or patients with chronic obstructive pulmonary disease (Granger et al., 2008; Reynoso-Roldán et al., 2012; To et al., 2012; Kowshik et al., 2014).

Blood flow is a critical factor inducing angiogenic sprouting, in the process of which HDAC1 was phosphorylated and translocated from the nucleus to cytosol. Similarly, phosphorylation and nuclear export of HDAC5 also mediated VEGF-induced angiogenesis (Ha et al., 2008). In addition to the effect of inducing angiogenesis during myocardial infraction (Yang et al., 2019), HDAC6 also improved repair stimulated by injury (Wu et al., 2016). By targeting the antiangiogenic microRNA-17-92 cluster, HDAC9 accelerated angiogenesis in ECs (Kaluza et al., 2013). In addition, HDAC5 played an important role in inhibiting expression of the genes participating in angiogenesis including CYR61, PVRL2, FSTL1, and Slit2 in patients with systemic sclerosis, which could be reversed by silencing of HDAC5 (Urbich et al., 2009; Tsou et al., 2016). Endothelial migration is a key step of angiogenesis, and both HDAC6 (Li et al., 2011; Birdsey et al., 2012) and HDAC7 (Mottet et al., 2007; Yu et al., 2014) could promote angiogenesis by regulating cell migration. On the contrary, it was shown that HDAC3 was a negative regulator of angiogenesis (Park et al., 2014). Treatment with TSA or SAHA suppressed HIF-1α, VEGF, VEGF receptors (VEGFR1 and VEGFR2), and the formation of capillary-like structures, but increased semaphorin, a VEGF competitor, in rat lungs and cultured human pulmonary microvascular ECs (Deroanne et al., 2002; Mizuno et al., 2011). SAHA could upregulate the WNT-inducible secreted protein 1, a matricellular molecule that accelerates angiogenesis in TNF-α-stimulated HCAECs (Wright et al., 2018). Importantly, the HDACi such as VPA (Jin et al., 2011; Wang et al., 2012) and SAHA (Jin et al., 2011; Wright et al., 2018) mainly exerted proangiogenic effect, whereas TSA (Williams, 2001) could also inhibit angiogenesis.

#### HDACs and Cell Differentiation Into ECs

When the endothelium is damaged or nearly denuded, the circulating or local resident stem or progenitor cells would differentiate into ECs to rescue the denuded ECs (Rinkevich et al., 2011). Rossig et al. (2005) found that inhibition of HDACs abolished the endothelial differentiation from adult progenitor cells by inhibiting the expression of homeobox transcription factors HoxA9, which is a regulator of eNOS, VEGF-R2, VE-cadherin, and EC maturation induced by shear stress. TSA improved H3K9 acetylation and downregulated HDAC1 expression in bone marrow progenitor cells, which were further treated to generate cardiac progenitor cells and were able to differentiate into myocytes and ECs in the infarcted mouse heart (Rajasingh et al., 2011). Furthermore, TSA induced EC marker VE-cadherin, von Willebrand factor (vWF), and Flk in VEGF-treated multipotent adult progenitor cells and induced the differentiation into ECs (Mahapatra et al., 2010). However, in the progression of stem cell-derived ECs differentiating into ECs, TSA showed an opposite effect. While VEGF induced the differentiation accompanied by increased activity of HDAC, TSA or silence of HDAC3 reduced the EC lineage marker, indicating that the HDAC3 positively regulated the differentiation (Xiao et al., 2006; Zeng et al., 2009). Moreover, HDAC6 was important for maintaining mechanical sensing in human induced pluripotent stem cell-derived ECs (Smith et al., 2018).

#### HDACs and Vascular Tone

Endothelium is crucial in regulating vascular tone to control the local cardiovascular function, especially by NO, which determines the endothelium-dependent relaxation and thus, to some extent, prevents hypertension and PAH (Pan et al., 2018). PAH results in the unnatural proliferation of PAECs, triggering enhanced pulmonary vascular resistance, as well as right ventricular failure. It is recognized that PAECs, pulmonary arterial smooth muscle cells, fibroblasts, and pericytes are involved in the pathogenesis of PAH (Rabinovitch, 2012). Furthermore, the factors secreted from PAECs such as fibroblast growth factor 2, IL-6, and ET-1 induce proliferation, migration, and vascular remodeling by influencing other cells in the development of PAH (Ricard et al., 2014). Kim et al. (2015) identified that MEF2 acted as the key *cis*-acting factor that regulated the expression of target genes contributing to pulmonary vascular homeostasis, such as microRNAs-424 and -503, connexin 37, connexin 40, KLF2, and KLF4, which were evidently decreased in PAECs isolated from PAH patients. This action could be abolished by nuclear accumulation of HDAC4 and HDAC5, causing inhibition of MEF2 transcription activity. Importantly, several HDAC isoforms were upregulated in isolated pulmonary arteries in a monocrotaline-induced PAH in rat models, and HDACi inhibited Nox expression and PAH markers in isolated pulmonary arteries (Chen et al., 2016). In addition, HDAC1 was involved in increased systolic blood pressure in mice by regulating eNOS expression and NO production, which could be inhibited by VPA (Won et al., 2014). The increased NO is the major vasodilator that reduces the vascular tone, which plays a critical role in maintaining arterial patency (Nishida et al., 1992). Several studies have shown that HDAC6 was crucial in impairing vascular tone. On the one hand, HDAC6 regulated chromatin remodeling and promoted ET-1 expression (Li et al., 2016). On the other hand, it was upregulated in AngII-treated aortas and HAECs, which induced cystathionine γ-lyase ubiquitination and degradation, and was implicated in AngII-induced hypertension (Chi et al., 2019, 2020). Moreover, PAH was significantly ameliorated in HDAC6^–/–^ mice. Similarly, HDAC8 was implicated in AngII-induced hypertension, and its selective inhibitor PCI34015 could reverse the effect and reduced vascular hypertrophy and inflammation (Kee et al., 2019). IGF-1 is a growth factor contributing to PAH in neonatal mice, through activating the AKT signaling pathway. Apicidin, an inhibitor of HDAC, suppressed pulmonary IGF-1/pAKT signaling pathway, which ameliorated right ventricular hypertrophy and vascular remodeling in lungs (Yang et al., 2015). And TSA could inhibit hypertension induced by abdominal aortic constriction in rats (Kang et al., 2015).

### HDACs Regulate VSMC Proliferation and Migration in Atherosclerosis

During the development of AS, VSMCs migrate to the subendothelium space and switch to a collagen-secreting synthetic phenotype (Doran et al., 2008) to form the fibrous cap, with mass of the foamy macrophages and cholesterol on the surface. Moreover, the proliferating VSMCs can also remodel the ECM by degrading the matrix components through increasing expression and activity of MMPs, which disrupts the fibrous cap of the plaque, causing plaque vulnerability (Shah, 1996).

Increasing evidence has shown the important role of HDACs in regulating VSMC proliferation and migration. Laminar flow enhanced HDAC1 activity and the interaction between HDAC1 and p53, which resulted in p21^*WAFl*^ activation in VSMCs (Zeng et al., 2003). Sun et al. (2020) found that HDAC1 was critical for the migration and phenotypic switch of aortic VSMCs. KLF4 and KLF5 acetylation in VSMCs was regulated by phosphorylated HDAC2, which was implicated in VSMC proliferation induced by retinoic acid receptor agonist (Meng et al., 2009; Zheng et al., 2011). HDAC4 also promoted VSMC proliferation and migration (Usui et al., 2014; Li et al., 2018b; Zhang et al., 2019), whereas interfering HDAC4 could inhibit the effect (Zheng et al., 2019). HDAC5 in VSMCs could be activated by AngII, a potent stimuli for VSMC proliferation (Pang et al., 2008). Moreover, Zhou et al. (2011a) showed that by regulating β-catenin translocation, splicing of HDAC7 induced SMC proliferation. In addition, HDAC2 and HDAC5 hypoacetylated histone H4 at the promoter site of α-smooth muscle actin and decreased the expression of marker genes of SMC differentiation induced by POVPC (Yoshida et al., 2008).

It is recognized that cyclic strain regulates phenotype switch, migration, and proliferation of VSMCs in the pathogenesis of AS (Haga et al., 2007). Cyclic strain induced the secretion of transforming growth factor β1 in VSMCs and the expression of contractile phenotype markers, such as smooth muscle protein 22α, α-smooth muscle actin, and calponin (Yao et al., 2014). It was shown that the cyclic strain induced the migration of VSMCs by up-regulating HDAC7 and down-regulating the levels of HDAC3/4. Tributyrin, a pan-inhibitor of HDAC, could suppress VSMC migration accompanied by reduced expression of HDAC7 (Yan et al., 2009). TSA could inhibit VSMC proliferation by inducing expression of p21^WAF1^, rather than p16^INK4^, p27^KIP1^, or p53, followed by cell cycle arrest through reducing Rb phosphorylation at the G1–S phase (Okamoto et al., 2006). It was reported that butyrate, a dietary HDACi, had the effect on arresting the proliferation of VSMCs. Furthermore, butyrate downregulated the G1-specfic CDKs including CDK4, CDK6, and CDK2 and induced the expression of CDK inhibitors, p15^INK4b^ and p21^Cip1^, leading to cell cycle arrest of VSMCs (Mathew et al., 2010). The authors also found that the effect of the butyrate was mediated by acetylating H3K and phosphorylating H3-serine10, in addition to dimethylation of H3K9 and H3K4 (Mathew et al., 2010). Besides, HDAC4 and HDAC5 was involved in vascular calcification (Abend et al., 2017; Choe et al., 2020) and inflammatory response in VSMCs (Lee et al., 2008), whereas HDAC1 (Liu and Khachigian, 2009) and HDAC5 (Pietruczuk et al., 2019) acted as a proinflammatory molecule in VSMCs. TSA could inhibit VSMC calcification (Azechi et al., 2013). All these studies revealed that the classical HDACs mediate the proliferation and migration of VSMCs, which could be blocked by HDACi.

### HDACs Are Involved in Macrophage-Derived Foam Cell Formation

Macrophage-derived foam cell formation is one of the major contributors to AS development. In the early stage of plaque formation, macrophages are mainly derived from the monocytes infiltrating into subintima (Ley et al., 2007). The macrophages stimulated by ox-LDL and its lipid components produce more inflammatory factors, such as IL-α, IL-β, IL-6, IL-18, and TNF-β (Poston, 2019), which trigger adhesion molecule secretion from ECs to attract more monocytes to differentiate into macrophages (Ley et al., 2007). By uptaking ox-LDL through binding to scavenger receptors such as CD36, macrophages became lipid-laden foam cells (Orekhov, 2018). MCP-1 expressed by VSMCs guides the foam cells to the atherosclerotic plaques (Tesfamariam and DeFelice, 2007). Unfortunately, when the foam cells underwent cell death (apoptosis or necrosis), the inner cholesterol components are released to the plaque. The released cholesterol, together with the collagen-secreted VSMCs and foam cells, contributes to the early plaque (Poston, 2019).

Treatment with TSA markedly upregulated the expression of CD36 in macrophages, which increased the uptake of oxLDL and accelerated AS (Choi et al., 2005). The lipid efflux, removing redundant cholesterol from macrophage, is a vital step in suppressing the development of AS (Li et al., 2010). ATP-binding transporters (ABCA1 and ABCG1) transfer the cholesterol from macrophage to lipid-poor apolipoprotein and high-density lipoprotein (HDL). Interestingly, inhibition of HDAC with TSA, ITF2357 (pan-HDACi), or RNA interference to silence genes of HDAC 1/2/3/6/8, respectively, increased histone acetylation and ABCA1/ABCG1 expression, which suppressed the accumulation of cholesterol in macrophage, and HDAC3 silence appeared the most effective (Van den Bossche et al., 2014). The precise role of HDAC isoforms and the pharmacological effects of HDACi in foam cell formation warrant further studies. Additionally, PPARγ is a transcriptional factor critically involved in lipid metabolism and possesses anti-inflammation properties (Vallée et al., 2019). Recent study showed that, by activating PPARγ through elevating acetylation of C/EBPα (CCAAT enhancer binding protein α), TSA increased ABCA1/ABCG1 expression and reduced TNF-α/IL-1β, which contributed to the inhibition of foam cell formation and atherogenesis (Gao et al., 2020).

### HDACs and Thrombus Formation

In the development of AS, both prothrombotic molecules and procoagulant molecules contribute to thrombosis formation. Tissue factor (TF) produced by plaque macrophages activates platelets through the extrinsic pathway to initiate thrombin signaling (Collot-Teixeira et al., 2007). In addition, ox-LDL is considered to be another extrinsic factor that activates the platelets through its scavenger receptors (Ivanciu and Stalker, 2015). Thrombin activates ECs to release vWF, a factor that stimulates platelets (Spronk et al., 2013). The activated platelets potentially stimulate the platelets in a self-perpetuation manner, which produces more thrombin (Spronk et al., 2013). Wang et al. (2007) found that the HDACi (TSA, MS-275, sodium butyrate, and VPA) suppressed the expression of TF and its bioactivity. TF can enhance the release of vWF that causes platelet adhesion to ECs. The transcription factor nuclear factor Y (NE-Y) interacts with both HDAC1 and histone acetyltransferase PCAF in vWF gene promoter, causing dissociation of HDAC1 from the complex and leading to more PCAF recruited to vWF promoter, thereby promoting vWF expression (Peng et al., 2007). t-PA is generated by ECs, acting as an antithrombotic factor that is involved in clearance of intravascular fibrin deposits in ECs. The expression of t-PA could be inhibited by HDAC through deacetylating histone H3 and H4 in the t-PA gene promoter (Lappas, 2012). Furthermore, treatment with TSA, MS-275 or VPA increased the t-PA expression (Larsson et al., 2013), respectively, whereas the effect of VPA could be suppressed by knockdown of HDAC3/5/7 (Larsson et al., 2012).

### HDACs Regulate Atherosclerosis in Human and Animals

Among the classical HDACs, HDAC9 is best known for its role in the development of AS. The *rs2107595* HDAC9 gene polymorphism leads to increased expression of HDAC9 in the internal carotid artery (Grbić et al., 2020) and plasma (Wang et al., 2016) and modulated gene expression in the blood of patients suffering from large vessel atherosclerotic stroke (Shroff et al., 2019). The polymorphism variant is significantly associated with AS (Prestel et al., 2019; Grbić et al., 2020) and may contribute to coronary AS and coronary artery disease risk (Wang et al., 2016). Consistently, HDAC9 deficiency in *ApoE*^–/–^ mice resulted in evidently reduced lesion size in aortas and less advanced lesions (Azghandi et al., 2015). Further studies showed that HDAC9 could activate inhibitory κB kinase and regulate atherosclerotic plaque vulnerability (Asare et al., 2020). Moreover, HDAC9 repressed cholesterol efflux by downregulating ABCA1, ABCG1, and PPARγ and alternatively promoted macrophage activation in AS (Cao et al., 2014). On the contrary, it was demonstrated that reduced expression of HDAC9 induced by miR-182 was implicated in increased levels of cholesterol, lipoprotein lipase, and proinflammatory cytokines in oxLDL-treated human THP-1 macrophages, which the author suggested might mediate the lipid accumulation in atherosclerotic lesions and thus promoted atherogenesis in *ApoE*^–/–^ mice administered with miR-182 agomir (Cheng et al., 2017). Moreover, it was found that HDAC1/2/3/4/6/11 were all upregulated in atherosclerotic carotid arteries and aortas from human and *ApoE*^–/–^ mice, respectively (Manea et al., 2020). By using aortic isografted model, Zampetaki et al. (2010) found that endothelial-specific knockdown of HDAC3 in aortas from ApoE^–/–^ mice robustly promoted atherosclerotic lesion formation. However, Hoeksema et al. (2014) found that HDAC3 was the only isoform upregulated in human ruptured plaques, and myeloid deletion of HDAC3 in LDL receptor knockout mice led to more stabilized atherosclerotic lesions, which might be mediated by phenotype shift of macrophages to be anti-inflammatory and less lipid accumulation. The distinct effects of HDAC3 in these two studies suggest the cell-specific function of HDAC3 during the development of AS. In ox-LDL-treated HAECs and aortas from *ApoE*^–/–^ mice, HDAC6 was upregulated, and its selective inhibitor tubacin prevented endothelial dysfunction and the development of AS (Leucker et al., 2017). Several HDACi such as TSA (Gao et al., 2020), metacept-1 (Vinh et al., 2008), BuA (He and Moreau, 2019), and SAHA (Ye et al., 2018; Manea et al., 2020) have been demonstrated to inhibit the development of AS.

### Potential Combination of BRD4 Inhibitors With HDAC Inhibitors

Recent years have witnessed the important roles of bromodomain and extra terminal (BET) proteins, which are readers of acetylated histones. The BET family comprises four members, including BRD2, BRD3, BRD4, and the testis-restricted BRDT, among which BRD4 is the most characterized and critically implicated in transcriptional regulation and atherogenesis (Lin and Du, 2020; Wang et al., 2020). Intriguingly, the BRD4 inhibitor RVX-208 (now called apabetalone) showed potent effect in increasing apolipoprotein A-I and HDL levels/particles and reducing AS (McLure et al., 2013; Jahagirdar et al., 2014; Gilham et al., 2016; Ghosh et al., 2017), and has been tested in clinical trials (Nicholls et al., 2016, 2018; Shishikura et al., 2019). Pooling completed phase 2 trial data that suggested its clinical benefits on reducing major adverse cardiovascular events in treated patients (Borck et al., 2020). However, a phase III trial (BETonMACE) completed recently showed that addition of RVX 208 to contemporary standard of care for acute coronary syndrome (ACS) did not significantly reduce major adverse cardiovascular events in patients with a recent (7–90 days) ACS, type 2 diabetes, and low HDL cholesterol (Ray et al., 2020). To date, little is known about the direct interaction of BETs and HDACs or the combinational effects of BETi and HDACi in vascular cells and AS. Nevertheless, because HDACs and BETs share many common targets and affect similar cellular activities, it will be very interesting to study their molecular interplay during AS development. Those studies will be very helpful to evaluate whether combinational strategy using specific HDACi with BETi or developing dual BET/HDACi would be promising for AS treatment, as the strategy is rational and has been suggested in cancer studies (He et al., 2020; Liu et al., 2020).

## Conclusion and Perspective

**FIGURE 2:**
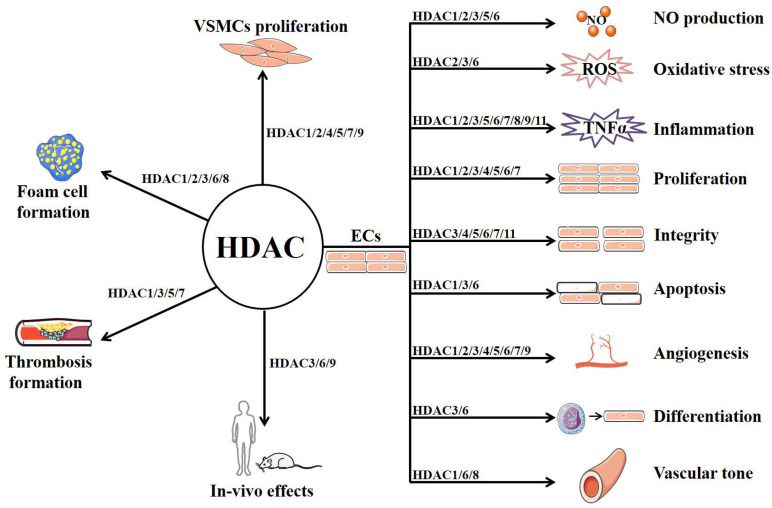
The potential mechanism of HDACs in atherosclerosis.

As a family of enzymes critically involved in chromatin remodeling and gene transcription, HDACs have multiple functions in ECs and other vascular cells that are implicated in AS (summarized in Figure 2 and Table 1) (Zhou et al., 2011b). Among the 11 classical HDACs, HDAC9 is the only subtype that has been well studied for its association with AS risk in both human and animals (Grbić et al., 2020). By contrast, little is known about the role of HDAC10 and HDAC11. With respect to other members, HDAC6 and HDAC8 mainly exhibit the role of mediating endothelial dysfunction and AS, whereas HDAC7 is mainly recognized as a protective isoform.

**TABLE 1:** Effect of HDAC members in endothelial (dys)function and atherosclerosis.

Class	Subtypes	Endothelial (dys)function and atherosclerosis (AS)													Included inhibitors	
		ECs									VSMCs	Macrophages	In vessel	*In vivo*		
							
		NO production	Oxidative stress	Inflammation	Proliferation	Integrity	Apoptosis	Angiogenesis	Differentiation	Vascular tone	proliferation	Foam cell formation	Thrombosis formation	Overall effects of AS	Selective inhibitors	Other specificity
I	HDAC1	−	*	−	+	*	−	+	*	−	+	+	−	*	*	Pan-HDAC: SAHA TSA ITF2357 Tributyrin Class I: MS-275 Butyrate Apicidin β-OHB Class I/II: VPA
	HDAC2	+	+	+/−	+	*	*	*	*	*	+	+	*	*	*	
	HDAC3	−	−	+	+	−	−	−	+	*	*	+	+	+/−	RGFP-966	
	HDAC8	*	*	+	*	*	*	*	*	−	*	+	*	*	PCI34051	
IIa	HDAC4	*	*	*	+/−	−	*	+	*	*	+	*	*	*	*	Pan-HDAC: SAHA TSA ITF2357 Tributyrin Class I/II: VPA Class IIa: TMP195
	HDAC5	+	*	−	+/−	−	*	+/−	*	*	+	*	+	*	*	
	HDAC7	*	*	+	−	+	*	+	*	*	+	*	+	*	*	
	HDAC9	*	*	+	*	*	*	+	*	*	+	*	*	+	*	
IIb	HDAC6	−	+	+	+	−	+	+	+	−	*	+	*	+	Tubacin/tubastatin A	
	HDAC10	*	*	*	*	*	*	*	*	*	*	*	*	*	*	
IV	HDAC11	*	*	−	*	−	*	*	*	*	*	*	*	*	*	Pan-HDACi

+, promoting; −, inhibiting; *, not clear.

As exemplified in this review, many studies have been performed to investigate the effect of HDACi in vascular dysfunction and AS. Although in most cases, HDACi could prevent the pathological process, some HDACi might exacerbate it. The reason might be attributed to the unspecific property of HDACi in either HDAC subtypes or tissues/cells, which might also limit their applications in clinic. Moreover, the dosage used in the experiments and the off-target effect would also affect the results. Interestingly, inhibitors specific to HDAC3 (Zhao et al., 2019), HDAC6 (Chen et al., 2019), and HDAC8 (Kee et al., 2019) have been found to be effective in regulating vascular homeostasis, but further studies are still required to evaluate the pharmacological and pharmacokinetic profiles. Moreover, as some HDACs exhibit protective effects, it is suggested that HDAC analogs might be designed and investigated, such as the HDAC7-derived peptide (Pan et al., 2018). Nevertheless, extensive studies are urgently needed to elucidate and validate the mechanisms of HDACs in vascular function and AS to develop the more specific and targeted HDACi with less toxicity or side effects. Furthermore, chronic toxicity studies and randomized controlled trials with optimized dosage are also required.

## Author Contributions

XC, YH, WF, YT, and HL wrote the manuscript. HL generated the illustration. AS revised the manuscript. SX conceptualized the manuscript, drafted the outline, and revised the manuscript. All authors contributed to the article and approved the submitted version.

## Conflict of Interest

The authors declare that the research was conducted in the absence of any commercial or financial relationships that could be construed as a potential conflict of interest.

## Abbreviations

ABCA1: ATP-binding cassette subfamily A1
ABCG1: ATP-binding cassette subfamily G1
AKT: protein kinase B
AMPK: adenosine 5 ′ -monophosphate (AMP)-activated protein kinase
AngII: angiotensin II
ApoE^–/–^: apolipoprotein E knockout
AS: atherosclerosis
Bcl-2: B-cell lymphoma-2
BH_4_: tetrahydrobiopterin
BuA: butyric acid
CAT: catalase
CD16: cluster of differentiation 16
cdk2: cyclin-dependent kinase2
cdk4: cyclin-dependent kinase4
cdk6: cyclin-dependent kinase6
COX-2: cyclooxygenase 2
CSE: cigarette smoke extract
CVD: cardiovascular disease
CYR61: cysteine rich angiogenic inducer 61
E2F1: E2F transcription factor 1
ECM: extracellular matrix
ECs: endothelial cells
ECV304 cells: human umbilical vein endothelial 304
eNOS: nitric oxide synthase
ERK 1/2: extracellular regulated protein kinases1/2
ET-1: endothelin 1
FSTL1: follistatin like 1
GLUT-1/4: glucose transport protein 1/4
H3K9: histone H3 lysine 9
HAECs: human aortic endothelial cells
HDAC6^–/–^: HDAC6 knockout
HDACi: histone deacetylases inhibitors
HDACs: histone deacetylases
HIF-1α: hypoxia inducible factor-1
HoxA9: homeobox A9
HPAECs: human pulmonary artery endothelial cells
HUVECs: human umbilical vein endothelial cells
ICAM-1: intercellular cell adhesion molecule 1
Id2: inhibitor of DNA binding 2
IGF-1: insulin-like growth factor 1
IKBα: NF-κB inhibitor
IL-10: interleukin 10
JNK: c-JunN-terminal kinase
KLF2/4: Krüppel-like factor 2/4
LPS: lipopolysaccharide
MAPK: mitogen-activated protein kinase
MCP1: monocyte chemoattractant protein-1
MDR1: multidrug resistance protein 1
MEF2: myocyte enhancer factor 2
MMPs: matrix metalloproteinase
MS-275: entinostat
NAD+: nicotinamide adenine dinucleotide
NE-Y: nuclear factor-Y
NF-κB: nuclear factor κB
NO: nitric oxide
Nox: NADPH oxidase
ONOO^–^: peroxynitrite
OSS: oscillatory shear stress
ox-LDL: oxidized low density lipoprotein
PAECs: pulmonary artery vascular cells
PAH: pulmonary arterial hypertension
PAI-1: plasminogen activator inhibitor type 1
PCAF: p300/CBP-associated factor
PPARγ: peroxisome proliferator-activated receptors
PVRL2: poliovirus receptor related protein 2
ROS: reactive oxygen species
SAHA: varinostat
sGC: soluble guanylyl cyclase
SIRT: sirtuin
Slc2a1: solute carrier family 2 member 1
Slit2: Slit homolog 2 protein
SMC: smooth muscle cell
SOD: superoxide dismutase
SP1: Specificity Protein 1
STAT1: signal transducers and activators of transcription 1
TF: tissue factor
TIMP: the tissue inhibitor of metalloproteinase
TLR4: toll like receptor 4
TNF-α: tumor necrosis factor α
t-PA: tissue-type plasminogen activator
TSA: trichostatin
VCAM-1: vascular cell adhesion molecule 1
VEGF: vascular endothelial growth factor
VEGFR: vascular endothelial growth factor receptor
VPA: valproic acid
VSMC: vascular smooth muscle cells
vWF: von-Willebrand factor.
